# Supplementary material for: Are carbon emissions trading and green financial instruments synergistic? -Comprehensive quantitative research based on content analysis
Source: PLoS One. 2024 Mar 7;19(3):e0298601. doi: 10.1371/journal.pone.0298601 (PMC10919601; doi:10.1371/journal.pone.0298601)
Supplement: S1 Data — (ZIP) [file pone.0298601.s001.zip › Data/地级市Z_F工作报告.zip!/║╙─╧/╔╠╟≡╩╨/└┤╫╘╣1⁄2╓┌║┼í╛┬φ┐╦╩2╛▌═°í┐_2009.docx]

各位代表：

现在，我代表市人民政府向大会报告工作，请予审议，并请各位政协委员提出意见。

一、过去一年的政府工作

2008年是极不平凡、极不寻常的一年，大事多、喜事多、难事多。在省委、省政府和市委的领导下，在市人大、市政协的监督支持下，市政府紧紧依靠全市人民，贯彻落实科学发展观，认真开展“思想大解放、经济大开放、全民大创业”实践活动，努力克服金融危机带来的不利影响，积极应对各种挑战和考验，团结进取，扎实工作，保持了经济社会平稳较快发展。

过去一年，是商丘经济持续发展的一年。预计全市实现生产总值930亿元，增长12％左右；地方财政一般预算收入31．5亿元，增长18．1％；地方财政一般预算支出118亿元，增长27．1％；全社会固定资产投资530．4亿元，增长32．3％；社会消费品零售总额287．8亿元，增长22．9％；金融机构年末各项存款余额602．7亿元，增长19．9％，贷款余额409．9亿元，增长12．2％。

过去一年，是商丘大事较多的一年。2008年是商丘建市10周年，我市成功举办了2008中国商丘国际华商文化节，依法查处了未来农业非法集资案，夺取了大沙河污染治理的胜利，开展了支援四川抗震救灾工作，举办了纪念改革开放30周年系列活动，经受了物价上涨、经济下滑、需求不足的金融危机考验，使全市人民激发了热情，鼓舞了斗志，增强了信心和勇气。

过去一年，是商丘喜事不断的一年。经济结构得到优化，一、二、三产业比例为27．3∶44．7∶28，历史上首次呈现二三一比例结构；粮食总产117亿斤，创历史最高纪录；中石油千万吨炼油项目落户我市，实现了国家重大项目建设的历史性突破；国电民权电厂一期两台60万千瓦机组投产发电；永煤一期年产50万吨甲醇项目竣工投产。这些都奠定了商丘未来发展的广阔空间。

过去一年，是商丘人民群众得到较多实惠的一年。城镇居民人均可支配收入11751元，增长15．6％，农民人均纯收入3750元，增长15．5％，城乡居民收入均实现两位数增长。

过去一年，是商丘捷报频传的一年。我市第五次荣获“全国双拥模范城”，荣膺“2008中国特色魅力城市200强”殊荣，被命名为“中国金融生态城市”，获得“全国粮食生产先进市”、“河南省绿化模范城市”称号，商丘的城市品牌形象和影响力都有了较大提升。

一年来，我们主要做了以下几个方面的工作：

（一）加大经济运行调节力度，促进经济平稳较快增长。面对国际国内复杂多变的经济形势，我们咬定发展不放松，采取了一系列促进经济平稳较快发展的政策措施。年初，积极顺应国家“防过热、防通胀”的“双防”宏观调控政策，加强经济运行调节，增加主要农副产品供应，稳定市场物价，通过“有保有压，调整结构”，确保了重点项目的资金需求。先后组织召开三次银企洽谈会，签约金额199亿元，到位158亿元，在全省处于第一位。迎峰度夏期间，针对煤炭价格上涨、电煤库存不足、电网稳供难度加大、发电企业亏损严重等问题，成立了全市煤电油运应急指挥中心，制定了保煤、保电、保运的工作措施，保证了工农业生产和人民生活对煤电油运的需求。第三季度以来，面对国际金融危机影响不断扩大、市场需求减弱、财政收入增幅减缓的局面，认真落实国家扩内需、保增长的政策措施，及时制定了支持工业经济发展、促进房地产业健康发展的具体办法，在第一时间筛选申报了一批重点项目，争取国家无偿资金5．31亿元。由于应对及时，措施有效，我市经济在困难局面下保持了平稳较快发展。对此，省委主要领导专门做出批示，给予充分肯定。

（二）着力转变发展方式，不断优化产业结构。坚持把调整结构作为经济工作的主线，在优化结构中转变发展方式，提高经济运行的质量和效益。工业经济结构进一步优化。围绕煤及煤化工业、铝及铝精深加工业、农副产品加工业、机械制造业等支柱产业上项目，着力推进资源型产业向精深加工发展，促进工业结构优化升级，支柱产业和重点企业保持了较快增长势头。全市52家大中型工业企业累计完成增加值177．3亿元，增长18．3％，占全市工业增加值的70％。永煤集团、神火集团分别位居“2008河南企业100强”第1位和13位，双双获得“改革开放30年河南省功勋企业”称号。十大工业集聚区累计建成标准厂房96万平方米，新建固定资产投资500万元以上企业300家，投产240家。自主创新能力增强。引导扶持企业加快技术创新和产品研发步伐，3家企业被认定为高新技术企业，3种产品被认定为高新技术产品，组建了4家省级工程技术研究中心。市政府与北京化工大学签署了科技合作框架协议。节能减排取得新成效。全面落实节能降耗和污染减排目标责任制，重点抓好高耗能行业和重点领域的节能减排。大力发展循环经济，8家企业被确定为资源综合利用企业。重点项目建设进展顺利。全年重点项目建设投资突破200亿元，112个重点项目竣工投产，计划开工的81个重点项目全部开工建设。服务业发展步伐加快。服务业实现增加值260亿元，占全市经济总量的28％。商丘演艺集团、王公庄农民画虎村，成为全国文化产业改革与发展的先进典型。全年接待国内外游客678．8万人次，创旅游收入11．34亿元，比上年增长35．3％和22．3％。

（三）扎实做好“三农”工作，深入推进新农村建设。围绕“粮食增产、农业增效、农民增收”的目标，落实各项支农惠农政策，“三农”工作保持良好发展势头。粮食生产稳定增长。新发展标准化种植小区20个，累计达到60个。夏秋两季粮食均获丰收，托市收购小麦56亿斤，比上年增加40亿斤，带动农民增收5亿元。林业生态建设成效显著。完成生态造林26．1万亩，各县（市、区）都达到了河南省县级平原绿化高级标准。畜牧业持续发展。新增规模化养殖小区88个，生猪、肉牛和羊存出栏率保持增长。全年肉类总产量48．2万吨，增长7．7％，奶类总产量21．4万吨，增长23％。农业产业化进程加快。各类农村经济合作组织发展到1400家，市级以上农业产业化重点龙头企业达到184家，其中国家级8家、省级27家。100个农畜产品通过了“三品”认证，65个无公害畜产品生产基地通过了省级认定。新农村建设扎实推进。广泛开展了“百村示范、千村带动、全面推进”、“市直单位驻村帮扶”、“百企帮百村”等一系列活动，涌现出了一批先进示范村。农村基础设施建设得到加强，新建沼气池4万座，修建改善农村公路1155公里，解决了26．38万人的饮水安全问题。农田水利基本建设成效显著，连续8年第15次荣获“红旗渠精神杯”。支农惠农政策全面落实。全市共落实粮食直补和农资综合补贴资金7．63亿元，补贴面积930．6万亩，补贴农户174万户。落实良种补贴、农机补贴、农村劳动力培训补贴1．29亿元，使农民群众得到了更多实惠。

（四）进一步深化改革开放，提高招商引资成效。按照科学发展观要求，创新体制机制，推进各项改革，扩大对外开放。国企改革和战略重组深入推进。中建材收购振华玻璃厂、南京雨润集团与福源集团、银河纺织与九天纺织合资合作取得实质性进展。神火集团与商电铝业集团、永煤集团与安化集团实施了战略重组。文化体制改革取得成效。实施了文化事业单位转企改制，商丘演艺集团的改革经验受到省委、省政府表彰和奖励。财政体制和水利工程管理体制改革步伐加快。深入推进国库集中支付制度改革，扩大了财政统发工资范围。5个县（区）完成了水利工程管理体制改革任务。招商引资和对外开放成效显著。新落地项目中，投资千万元以上的314个，实际到位资金69．4亿元，投资3亿元以上的11个，投资总额72．6亿元。新批外商投资企业7家，合同利用外资1．37亿美元，增长27％。实际到位外资7280万美元，增长6％。完成进出口总额3．6亿美元，增长13％。组织开展了市庆10周年、“5＋2”经济合作和区域定向招商活动。2008中国商丘国际华商文化节，省委书记徐光春同志给予肯定，称赞本次文化节“规模大、收获大、影响大。”

（五）统筹城乡协调发展，不断改善人居环境。以创建国家园林城市为载体，不断加强城市环境综合整治工作，着力提高城市活力和竞争力。城市规划体系不断完善。编制完成了《商丘市城市发展战略规划》、《商丘城市总体规划》，重点加强了中心城区住房建设、道路设计、河网水系、旧城改造、工业集聚区布局以及新农村建设等专项规划。城镇化水平进一步提升。全市城镇建成区面积达到352．8平方公里，中心市区93．2平方公里，全市城镇化率33．5％，比上年增长2个百分点。建成了宋城公园、金世纪公园、神火大道绿化精品段等一批城市公园绿地，城市绿地率达到31．2％。城市建设管理工作不断加强。完成了运河上游万吨污水处理厂和香君路建设，开工建设了市污水处理厂二期和睢阳北路、长江东路、长江西路。污水管网、供热、供气、供水进一步完善，6个县污水处理厂、垃圾处理场如期建成并投入运行。完成“空心村”整治125个。实行了城区门前“三包”责任制和领导干部牵头的“路长”单位负责制。开展了国有土地使用权出让情况专项清理，对闲置土地处置政策执行情况进行了监督检查。加大违法用地、违法建设整治力度，立案查处土地违法案件148起。房地产业健康发展。全市房地产开发投资完成54．4亿元，增长104．3％，其中住宅投资45．9亿元，增长103．2％。

（六）为群众多办实事好事，推进和谐商丘建设。十件实事得到了全面落实，全年用于办实事的总投入达到38亿元。一是大力发展教育事业。投入3000万元，在市区新建4所中小学，缓解了部分区域入学难状况。加快发展职业教育，加大了对农民工培训和实用技术人才培养力度。商丘师院、商丘医专和商丘科技学院通过教育部人才培养评估，夏邑职教中心被评为国家重点中等职业学校。二是推进文化事业和文化产业发展。实施农村电影放映工程，放映公益电影5万多场，广泛开展送戏下乡活动，共演出1800多场。加强文化名市建设，开展文化信息资源共享工程和乡镇文化站建设，完成农家书屋工程280家。王公庄获得“中国艺术之乡”称号。大型古装豫剧《李香君》荣获河南文华大奖，《钟鸣钟庄》获省“五个一”工程优秀奖。三是提高公共卫生应急水平。加强农村卫生基础设施建设，完善城市社区卫生服务网络，疾病预防控制和公共卫生应急能力不断增强。艾滋病防治工作取得明显成效。高度重视“问题奶粉”事件和手足口病防治工作，迅速反应、科学防控，有效处置了这两起突发事件，确保了无死亡病例发生。四是进一步完善社会保障体系。城镇新增就业9．37万人，实现再就业3．6万人，城镇登记失业率3．7％，低于省定控制目标。全年实现劳务输出218万人，比上年增加16．8万人，创劳务收入140亿元。筹措廉租住房保障资金9740万元，享受补贴家庭1．52万户。开工建设经济适用住房36．8万平方米，竣工19．9万平方米。启动了商丘市创业孵化园区的建设工作，建成2．5万平方米的标准化厂房。加强城镇居民基本医疗保险工作，新型农村合作医疗覆盖面进一步扩大。城乡低保、农村五保对象供养标准以及五保对象集中供养率不断得到提高。五是加强平安商丘建设。开展了信访疑难案件大督查和“学法用法，做守法公民，建和谐商丘”活动，全国“两会”和北京奥运会期间，我市没有发生赴京集体上访，受到省委、省政府表彰。开展了“百日安全活动”和安全生产专项督查行动，安全生产形势平稳。加强食品药品监管，确保了人民群众的健康安全。强化社会治安综合治理，保持了社会大局稳定。六是援建四川灾区工作成效显著。四川汶川地震发生后，全市上下情系灾区、踊跃捐助，累计捐赠款物4683万元。先后派出卫生、消防、建设等7支队伍600多名人员，赶赴第一线开展抢险救灾和对口援建，建设了419套过渡安置房及配套设施，解决了1800多名灾区群众的临时住房问题。市援川救援队获得中华全国总工会“抗震救灾重建家园工人先锋号”荣誉，市公安消防支队被公安部授予“抗震救灾先进集体”称号。

（七）开展“两转两提”活动，加快推进政府工作创新。深入开展转变政府职能、转变工作作风、提高行政效能、提高公务员素质活动，对261项行政许可和非行政许可项目进行了清理，实施了行政事业性、经营服务性“两项收费”专项清理，取消130项收费项目。加快推进阳光政务，全市所有乡镇（办事处）建立了便民服务中心，形成了市、县、乡三级行政服务网络。加强社会主义民主法制和精神文明建设，认真贯彻《全面推进依法行政实施纲要》，强化了对行政执法主体的执法监督。廉政建设和反腐败工作继续深化。自觉接受人大的法律监督、工作监督和政协的民主监督以及社会各界的监督，认真听取民主党派、工商联和无党派人士的意见，充分发挥工会、共青团、妇联等人民团体的作用，累计办结人大代表议案、建议、政协委员提案733件，办结率100％。社会管理和公共服务工作不断加强，国防教育、民兵预备役建设工作取得新进展，人口和计划生育工作被授予河南省先进单位称号。

各位代表！

一年来，在挑战大、困难多的情况下，取得的成绩确实来之不易。这是全市上下和衷共济、共渡时艰的结果，凝结着全市人民的心血和智慧，得益于社会各界的支持和帮助。在此，我谨代表市人民政府，向辛勤工作在各行各业的广大工人、农民、知识分子、干部、人民解放军指战员、武警官兵、公安民警和离退休老同志，向所有关心支持商丘建设与发展的各界人士，表示衷心的感谢和崇高的敬意！

回顾总结一年来的工作，我们也清醒地认识到经济社会发展和政府工作中还存在许多困难与问题：

——保持经济快速增长的压力较大，受国际国内经济发展环境变化的影响，从去年7月份开始，我市一些经济指标增长速度逐步回落，部分企业生产经营困难，市场需求下降，项目建设进度受到影响。

——经济发展水平还不高，调整产业结构和转变发展方式的任务十分艰巨，节能降耗压力不断加大。

——农业生产基础薄弱，防灾抗灾能力不强，农民持续增收难度增大。

——财政收支矛盾突出，收入增长明显放缓，减收增支压力较大。

——政府职能转变还不到位，应对突发事件的能力需要尽快提高，城市管理工作亟待加强。

——少数政府工作人员服务意识和依法行政观念不强，推诿扯皮、办事效率低下现象仍然存在，投资环境需进一步改善等。

对此，我们将采取更加有效的措施，认真加以解决。

二、2009年政府工作总体要求和奋斗目标

2009年可能是新世纪以来我国经济发展最为困难的一年，我们将面临着严峻的挑战和考验。国际金融危机导致世界经济增长明显减速，国际经济出现了整体下滑趋势，对我国实体经济的影响还在进一步加深，国内经济增长的不确定因素日益增多，防止经济下滑已成为经济运行中的突出矛盾。这场上世纪大萧条以来最严重的国际金融危机还在扩散和蔓延，危机尚未见底。受其影响，我市经济运行下行压力明显加大，经济领域的矛盾和问题日益显现，企业有效需求不足的矛盾突出，开工不足，效益下降，有些企业出现了停产半停产；农副产品价格回落，农民工回流，城镇新增劳动力规模扩大，就业形势严峻。商丘作为一个煤电铝占主导地位的产业结构受冲击较大，保增长任务十分繁重。

2009年也是蕴含发展机遇的一年，我们既要有危机意识，更要有机遇意识，不仅看到困难问题，还要看到有利条件：首先是国家实施积极的财政政策和适度宽松的货币政策，出台了一系列扩内需、保增长的政策措施，并将投入4万亿元的配套资金，这为我们争取国家财政支持提供了机遇。二是十七届三中全会制定了扶持“三农”的优惠政策，省委、省政府对黄淮四市继续支持，河南省实施“8511”投资促进计划，将带动新开工项目投资1万亿元以上，有利于我市加大投入保增长，争取项目增后劲，这些利好因素可以帮助我们解决自身解决不了的问题。三是从我市现实看，经过多年的积累，商丘已具备一定的发展基础，随着我市列入国家重大项目的投产，随着省里新开工项目的推进，随着规模企业的不断增加，随着综合交通体系的构建，资源优势、区位优势、产业优势和政策优势将进一步得到发挥，发展动力将日益增强，必将为大投入、大开发、大开放创造有利条件。四是当前我市正处于工业化、城镇化、农业现代化的关键发展时期，基础设施建设、产业发展、居民消费等方面都有巨大的空间，建设成本较低，制约因素较少，有利于我们在应对危机和困难中抢抓机遇，促进发展。因此，挑战和机遇同在，困难与希望并存，我们要把宏观政策调整和国家扩大内需的措施作为抓投入、上项目的机遇，作为调整优化产业结构的机遇，作为招商引资的机遇，做到信心不懈，脚步不停，准确把握形势，抓住难得机遇，扎实做好工作，变压力为动力，化危机为转机，在困难中开拓新的发展空间，在新一轮竞争中实现异军突起。我们对做好今年各项工作，实现全年奋斗目标充满信心。

今年政府工作的总体要求是：全面贯彻党的十七大和十七届三中全会、省委八届九次全会和市委三届八次全会精神，以科学发展观统领经济社会发展全局，把保持平稳较快发展作为经济工作的首要任务，把改善民生、促进和谐作为政府工作的出发点落脚点，把优化服务、务实清廉作为自身建设的根本要求，使谋发展、保增长、调结构、促和谐更好地结合起来，着力加大投资拉动和扩大对外开放，着力转变发展方式和调整经济结构，着力保障和改善民生，着力突出服务和优化环境，扎扎实实做好各项工作，确保经济社会发展目标全面完成，确保发展、民生、环境取得新成效。

今年经济社会发展的主要预期目标是：全市生产总值增长11％左右；地方财政一般预算收入增长9％；全社会固定资产投资增长23％；社会消费品零售总额增长15％；进出口总额增长10％，实际利用外商直接投资增长14％；城镇居民人均可支配收入增长7％，农民人均纯收入增长6％；人口自然增长率、城镇登记失业率和单位生产总值能耗降低率等控制在省定目标以内。

面对当前形势，做好今年的政府工作，实现今后的发展目标，必须认真把握以下原则：

——更加注重科学发展。形势越是严峻，落实科学发展观的要求越加迫切。要坚持以科学发展观为指导，全面落实保增长、扩内需，调结构、快转型，重民生、促和谐的各项政策措施，遵循规律，遵守纪律，把应对挑战与科学发展的要求紧密结合起来，用科学谋划实现趋利避害，用科学发展破解现实难题，在克服困难中实现新的发展，在应对挑战中实现新的跨越。

——更加注重项目主导。坚定实施项目带动战略，深入开展“项目建设年”活动，把项目建设作为抓机遇、保增长的根本举措，以项目论英雄，以项目保增长，以项目促发展，对重大项目定领导、定人员、定任务、定时限、定责任、定奖惩，千方百计争取国家投资项目，全力以赴抓好招商引资项目，用项目突破瓶颈制约，用项目推动产业升级，用项目促进跨越发展。

——更加注重增创优势。围绕建设与郑汴相呼应的区域性中心城市的目标，发挥资源禀赋优势，加快产业发展及升级步伐，进一步强化交通枢纽功能，着力构建豫东现代化城市的基本框架，努力在产业发展、区位交通、城镇建设上增创新优势，推动结构调整，拓展服务功能，集聚资源要素，不断提高辐射周边的综合能力，提升在全省的地位形象。

——更加注重以人为本。越是经济困难时期，越要高度关注民生，不断改善民生，促进社会和谐。想问题、办事情、做决策，坚持民生为重的观念，尊重群众意愿，考虑群众利益，关心弱势群体，解决好人民群众关注的教育卫生、就业创业、社会保障、住房行路、环境保护、社会稳定等切身利益问题，让人民群众得到更多实惠，使改革开放成果由广大民众共享。

——更加注重优化环境。能否创造良好的投资环境，关系到中央保增长政策的贯彻落实，关系到商丘对资本、技术、人才的吸引力，关系到我市工商企业的生存发展。只有不断优化发展环境，才能打造新的发展平台，才能在新一轮发展中抢占先机，赢得主动。以环境吸引项目、吸引资金，以环境扶助企业、渡过难关，以环境聚集人气、树立形象，应成为每一个公民的自觉行动。

三、转变发展方式，促进经济平稳较快发展

应对今年的经济形势，必须把转变发展方式，推动结构调整作为主攻方向，使扩内需、保增长与调结构、促转型更好地结合起来，突出重点，统筹兼顾，着力保持经济平稳较快发展。

（一）强力推进新型工业化进程。企业是经济之源、发展之本。必须把保持企业健康运行作为最现实、最重要、最紧迫的任务，重点抓好经济运行调节，做大做强骨干企业，增强自主创新能力，提高工业经济整体水平。加强工业经济运行调节。开展好“企业服务年”活动，认真分析工业经济运行中的新情况、新问题，广泛听取企业对政府工作的要求和建议，及时制定服务企业的具体措施和办法，分类指导，开展帮扶，千方百计帮助企业应对危机，走出困境，努力争取资金不断链、生产不停工、市场不丢失、职工不下岗。要加大对企业的扶持力度，完善信用担保体系，落实企业税收优惠政策，切实减轻企业负担。培植壮大骨干企业。实施“大型企业增规模、小型企业进规模、新上项目促达产”工程，扶持一批中小企业上规模，增强龙头骨干企业对经济的支撑带动能力，新增规模以上工业企业100家。着力培育主导产业。突出发展煤和煤化工产业链，打造煤化工基地，争取永煤集团煤化工基地二期年产50万吨甲醇、40万吨醋酸项目尽快开工建设。延伸铝产品深加工链，打造铝加工基地，开发铝深加工产品。打造加工制造业基地，以宇畅挂车、宗庆摩托、淮海铸造、德信集团等企业为基础，积极引进专用汽车、挂车、摩托车、汽车零部件加工项目。提升农副产品的精深加工度，推动农副产品加工业由资源数量型向质量效益型转变。大力推动自主创新。建立激励机制，鼓励技术创新和科技成果产业化，促进创新成果直接向现实生产力转化。推进企业工程技术研究中心建设，增强企业的自主创新能力和核心竞争力。深化科技合作与交流，加强与中科院、北京化工大学等科研院所、高等院校在科技合作、成果对接、人才培养等方面的深层次合作。继续深化企业改革。鼓励优势企业强强联合，加快推进神火集团与商电铝业集团电解铝资源战略重组，实现优势互补，做大做强电解铝产业。鼓励优势企业兼并重组困难企业，推进南京雨润重组福源集团，充分利用有效资产，稳定生产和就业。鼓励关联产业、关联企业和上下游产品联合重组，推进海泰公司与冰熊冷藏车的重组工作，提高规模效益。引导企业苦练内功，优化产品结构，提高产品质量，增强市场竞争力和抗风险能力。加快工业集聚区建设。充分利用省委、省政府支持黄淮四市加快发展的优惠政策，明晰产业定位，引导项目入园，支持企业裂变入园发展。加强园区基础设施建设和标准化厂房建设，确保每个县（市、区）工业集聚区新增标准厂房2万平方米以上，努力在发展主导产业、提升配套能力、完善设施功能方面取得突破。鼓励支持非公有制经济发展，加大对中小企业的扶持力度。每个县（市、区）都要建立一个以政府为主导、注册资金1000万元以上的担保公司。支持龙头企业、行业协会设立担保公司，提升信用担保能力，努力满足中小企业资金需求。

（二）强化重点项目建设。扩大投资是拉动经济增长的主要动力，必须紧紧抓住这个“牛鼻子”。今年全市固定资产投资确保达到652亿元，初步安排重点建设项目197个，其中续建项目92个、新开工项目93个、前期工作项目12个。工业及能源项目。围绕培育工业主导产业，安排项目95个，总投资610亿元，今年计划投资110亿元。重点抓好中石油（商丘）千万吨炼油厂、商丘230万千瓦热电厂、神火集团60万千瓦发电机组、国电民权电厂二期260万千瓦发电机组、两个500千伏输变电工程、阳光铝材年产15万吨铝板带一期5万吨生产线建设，促进结构调整、产业升级。交通项目。围绕建设综合交通体系，安排项目13个，总投资290亿元，今年计划投资38亿元。重点抓好邢商永地方准轨铁路、商周高速公路二期、沱浍河航运开发、郑徐客运专线、商丘军民合用机场等项目建设。农村基础设施建设项目。围绕大型商品粮基地和新农村建设，安排项目19个，总投资31亿元，今年计划投资8亿元。重点抓好大型商品粮生产基地、黄河故道生态治理、大中型水库除险加固、农村大型种植养殖小区等项目建设，改善农村生产生活条件。城市基础设施建设项目。围绕建设现代城镇体系，安排项目51个，总投资98．1亿元，今年计划投资48亿元。重点抓好城市道路修建改造、供排水管网建设、中水回用、工业污染点源治理等项目，完善城市功能。市场物流、社会事业项目。围绕加快发展社会事业，建设区域性物流中心城市，安排项目19个，总投资15亿元，今年计划投资9．5亿元。重点抓好物流园区、市中医院综合病房楼、县级中医院改扩建、初中校舍改造、医疗卫生保障体系建设，更好地服务群众，改善民生。

（三）加快农村经济发展步伐。贯彻落实党的十七届三中全会和今年中央一号文件精神，制定推进土地流转、加强粮食核心区建设、支持返乡农民工创业就业、发展现代农业的政策措施，促进农民增收，加快新农村建设步伐。高度重视粮食生产。组织实施粮食核心区建设，加大资金投入，建设旱涝保收田，加强优质粮工程建设、高产田开发和中低产田改造，提高农业综合生产能力，确保国家粮食安全，稳定增加农民收入。大力发展现代农业。坚持以工业的模式发展现代农业，围绕面及面制品加工、畜牧养殖及肉奶加工、食用菌、林果及加工、林板浆纸一体化、棉纺主导产业，推行标准化种植，发展规模化养殖，建设一批优势农畜产品基地。重点扶持一批年销售收入超亿元、具有竞争优势和带动能力强的农业产业化龙头企业，壮大产业规模，加快形成产业优势。健全社会化服务体系，积极发展多种形式的农民专业合作组织。认真落实各项支农惠农政策，增加农民收入，切实保障农民利益。加大财政扶持力度，健全农业保险机制，促进现代农业持续发展。改善农村生产生活条件。以引黄灌区节水改造、病险水库加固等为重点，加强农田水利基本建设，提高抗御自然灾害的能力。改善农业生态环境，巩固林业生态市创建成果，加快发展速生丰产林和名特优新经济林。加强农村安全饮水、沼气、道路、电力、通讯、有线电视、自来水、卫生室等基础设施建设。抓住土地利用总体规划、城乡规划和产业集聚区规划编制的机遇，做好新农村建设规划编制工作，优化土地利用结构和布局。深化农村改革。在不改变土地集体所有制性质、不改变土地用途、不损害农民土地承包权益的前提下，按照依法、自愿、有偿的原则，探索土地流转办法，加快转变农业发展方式。改善和创新农村金融体制，扩大信贷支持规模。加快农村集体林权制度和水利工程管理体制改革步伐，解放和发展农村生产力。

（四）进一步提升城市化水平。按照省委、省政府构建现代城镇体系的总体要求，围绕建设与郑汴相呼应的区域性中心城市，加强基础设施建设，创建国家园林城市，打造宜居环境。做好规划编制工作。坚持“规划即法、执法如山”的观念，建立健全规划实施机制和监督机制，实现中心城区近期建设用地范围内控制性详规全覆盖。加强县城规划编制，全部完成县（市）总体规划修编工作，编制商虞一体化发展战略规划。加强对乡村规划的督促指导，完成村级规划编制60％。高起点编制城乡一体化发展规划，围绕城乡空间和产业布局等关键领域，高标准做好控制性详规工作。加强基础设施建设。加快推进阏伯路、睢阳北路、神火大道、南京路、文化路、凯旋路等城市道路的新建、改造和绿化、亮化工程。搞好林业生态市建设，创建国家级园林城市。实施包运河两岸的绿化、美化、亮化，加快城市供（排）水、供热、天然气、西气东输管网工程二线、长江路包河桥和运河桥建设进度。加强城市卫生基础设施建设，迎接省级卫生城复核验收。完善优惠政策，大力推进道北旧城开发改造。进一步加快县城和小城镇建设步伐。深入推进精细化管理。围绕整治市容环境、违法建筑、交通秩序，提升绿化、美化、净化、亮化水平，着力构建治理城区脏、乱、差的长效机制。在环卫保洁、绿化美化、市政设施管护等方面，实行市场化运作，解决管理队伍和管理投入问题。继续搞好违法用地、违法建设专项治理，有效遏制违法建设行为。巩固门前“三包”及“路长”负责制整治成果，对背街小巷加强环境卫生、市场和交通秩序的综合整治，加大执法力度，形成联动机制，整合城管资源，创新城管理念，把城市管理覆盖延伸到城市的各个社区和街道，切实改善城市环境，提升管理水平，优化人居环境。加强文明城镇创建工作，提高居民文明素质，塑造商丘良好形象。强化资源节约和环境保护。实行严格的节能减排工作责任制、问责制和“一票否决”制，严把项目准入关。对新上项目认真落实环评和“三同时”制度，决不能引进高耗能、高污染项目。加强耕地保护，落实耕地保护行政首长负责制，严格执行土地利用总体规划和年度计划。加大土地整理开发、黏土砖瓦窑场土地复垦、“空心村”整治力度，继续开展市区和农村个人建房用地清查处理。

（五）大力发展现代服务业。把现代服务业的发展，作为扩大内需、增创优势的战略措施，切实发挥消费拉动经济增长的作用，着力增强区域性中心城市的辐射带动力，更好地满足人民群众的消费需求。促进商贸物流业做大做强。发挥好区域性物流枢纽城市的优势，加快发展高速公路、地方铁路、民航和水运，建立大交通，促进大流通。推进豫东综合物流园区项目前期工作，让更多的物流企业入驻商丘。以商丘农产品中心批发市场、中原车城等为依托，建设大型物流枢纽。继续实施万村千乡市场工程、“家电下乡”工程，建立和完善农村商贸流通网络，扩大农村消费市场。保持房地产业健康发展。认真落实促进房地产业健康发展的政策，鼓励开发企业建设中小套型、中低价位住房，使大多数居民能够买得起房，让特困群体和困难家庭享受廉租房优惠政策。突出抓好旧城改造和城中村改造，集约节约用地，提高开发水平。大力发展楼宇经济，鼓励房地产开发商和物业公司开发包装、营销租售楼宇，积极引进国内外知名企业（集团）总部或品牌总代理机构进驻商丘，推进楼宇经济发展。加快文化旅游业发展步伐。推进文化名市建设，叫响“华商之都”城市品牌，进一步提升商丘的文化影响力和竞争力。深化文化体制改革，推广演艺集团的改革经验，搞好公益性文化事业单位内部管理体制改革，推进艺术院团、影剧院和文化演出公司、电影公司改制重组为龙头企业和企业集团，鼓励多出精品力作。加速推进旅游资源优势向经济优势转变，围绕商丘古城、芒砀山景区、黄河故道游览区、王公庄画虎村、“华商之都”城市品牌等旅游资源做文章，加大开发、包装、推介力度，发展不同特色的文化游、乡村游、生态游、红色游、城市游、企业游等，打造精品旅游线路，增强软实力。提升现代金融业发展水平。鼓励更多的商业银行和证券公司及非银行金融机构，在我市开办分支机构。加强金融生态环境建设，防范金融风险，实现互利共赢，共同发展。

（六）深入开展招商引资。把扩大开放、招商引资作为应对危机、抢抓机遇，增创优势、持续发展的重大战略措施，持续开展招商攻势，大力组织引资活动，严格目标奖励措施，努力提高招商引资工作水平。拓宽招商引资视野。把专项招商和重大节会作为信息发布、促进招商的重要手段，集中发布项目，大力推介商丘，广交商界朋友，开拓合作交流空间。扩大招商引资渠道。一方面围绕国家产业政策，认真筛选项目，引进政策性资金；另一方面，充分发挥人文、资源、区位等优势，加快承接国际及沿海发达地区产业转移，千方百计吸引民间资本，引导更多的社会资金在商丘投资兴业。突出招商重点。继续把引进大项目作为招商引资的一项重要指标进行考核，努力引进综合实力强的战略投资者，着力加强以现有企业为载体的招商引资工作，对引进投资规模超亿元的重大工业项目，按照“一事一议”原则重点推进、重点服务、重点奖励。完善激励机制。对招商项目从投资意向到正式签约，从办理手续到开工建设，从开始施工到竣工投产，都要有专人负责，全程跟踪服务，认真解决项目在落地、建设和生产经营中遇到的各种困难和问题。继续对各县（市、区）和各单位招商引资目标进行量化分解，落实任务，把招商引资成果作为检验干部能力、工作政绩的一条硬指标，加强考核，兑现奖惩。

（七）着力营造环境树形象。营造良好的法制环境。加快推进依法治市进程，推进政务公开、村务公开、厂务公开，扩大基层民主。加强法制宣传教育，提高公民的法律素质，自觉遵纪守法。营造和谐的平安环境。切实健全应急管理机制，提高预防和处置突发事件能力，最大限度减少突发事件发生。切实抓好信访和社会调解工作，及时将矛盾纠纷化解在萌芽状态。营造严明的政务环境。深化行政审批制度改革，简化审批程序，规范审批行为，提高行政审批效率。加强对公务员的教育管理，努力建设一支人民满意的公务员队伍。营造宜人的生态环境。加大林业生态市和国家园林城市创建力度，认真落实节能减排责任制，严格环保责任追究制，加强造林绿化、水土保持、环境保护、城镇建设与管理工作，发展循环经济，改善人居环境。营造特色的人文环境。加强社会主义核心价值体系建设，深入进行社会公德、职业道德、家庭美德、个人品德教育，推动群众性精神文明创建活动蓬勃开展，铸造商丘诚实守信品牌形象。加强文化遗产和优秀民俗文化的保护与开发利用，提升商丘文化品牌的影响力。

四、大力发展社会事业，着力保障和改善民生

坚持以人为本、执政为民，多办利民之事，尽好安民之责，让广大人民群众共享更多改革发展成果。

大力发展教育、文化、卫生等社会事业。提高农村义务教育学校生均公用经费标准，中学由375元提高到500元，小学由230元提高到300元。继续对农村义务教育阶段学生免除学杂费、免费提供教科书，对农村寄宿制学校家庭经济困难学生补助生活费，对城市义务教育阶段的学生免除学杂费。加快中等职业教育发展步伐，建立市级职教中心，各县（市、区）完成新建或改建一所中等职业学校。完成33所农村初中校舍改造任务。积极发展文化事业。加强农村文化建设，继续实施广播电视村村通、文化信息资源共享、县级文化馆、图书馆、乡镇综合文化站和村文化大院建设、农村电影放映、农家书屋等文化惠农工程，广泛开展送电影、送戏曲下农村、进社区活动，丰富群众文化生活。

切实做好商丘古城和隋唐大运河申遗工作。推进卫生事业全面发展，健全市、县、乡、村四级医疗卫生服务网络。完善重大疾病防控体系和突发公共卫生事件应急机制，认真落实重大疾病防治措施。坚持不懈做好人口和计划生育工作，完善利益导向机制，稳定低生育水平，提高出生人口素质。

完善就业和社会保障工作。加强对“4050”人员、城市低保对象就业帮扶，认真落实零就业家庭和就业困难对象援助计划。重点做好返乡农民工和大学毕业生的就业、创业工作，企业要统筹安排，在困难时期力争不减员、不放假。加快完善城乡社会保障体系，继续扩大城乡居民养老保险覆盖面，提高城市低保和农村五保对象供养标准，完善城乡最低生活保障和城乡医疗救助制度，提高城乡医疗救助水平。

推进全民创业。完善和落实促进全民创业的政策措施，形成有利于各类市场主体创业的体制机制，支持城乡居民、返乡农民工、复员退伍军人和大中专毕业生自主创业。突出抓好农民技能培训，提高就业能力，建立健全返乡农民工就业帮扶机制，鼓励有技术、有资金、懂管理的返乡农民工自主创业。鼓励引导非公有制经济发展，支持非公有制经济进入公用事业、基础设施以及社会事业等领域。扶持重点民营企业做大做强。

加强社会管理和公共服务。进一步健全利益协调、诉求表达、矛盾调处、权益保障机制，把群众利益诉求纳入制度化、规范化、法制化轨道，切实维护土地征用、房屋拆迁、企业改制中的群众利益，提升社会和谐度。做好安全工作，坚决遏制重特大安全事故，强化食品药品监管，保障人民群众生命和健康安全。

巩固发展军政军民关系。关心和支持驻商解放军、武警部队建设，加强国防后备力量建设，健全国防动员体系，抓好民兵预备役和人民防空工作。深入开展双拥共建活动，巩固“全国双拥模范城”创建成果。认真落实军转干部、复员退伍军人优抚和安置政策，在新的起点上实现军政军民工作的新跨越。

办好十件实事。今年集中力量，继续为全市人民办好十件实事：

1．为城镇低收入家庭提供住房保障，今年新改建廉租房10万平方米。

2．提高城乡居民收入，企业退休人员基本养老金人均月增加100元以上；农村计划生育奖励扶助金由每人每年600元提高到840元；农村五保对象集中供养标准由每人每年1500元提高到1600元；完成130个贫困村整村推进扶贫开发建设任务，解决12万贫困人口温饱和脱贫问题；提高优抚对象、建国前老党员抚恤和生活补助标准。

3．在市区新建、改扩建4所中小学，缓解入学难和大班额问题。开工建设市儿童福利院和市救助管理站。

4．将全市大中专在校生全部纳入城镇医疗保险范围，城镇居民医疗保险覆盖面达到100％；完善新农村合作医疗制度，新农合参合率提高到91．5％；创建“白内障无障碍市”，免费为2万例白内障患者实施复明手术。

5．加快市创业孵化园区建设步伐。重点抓好返乡农民工的培训就业，筹集5000万元，免费培训农民工10万人。

6．投入2．9亿元，建设县乡公路300公里，改造危桥3000延米。继续实施农村安全饮水工程，解决30万人安全饮水问题。新建沼气池5万座。实施农田机井通电工程。

7．完成国家园林城市创建任务，加快林业生态市建设，改善人居环境。完成阏伯路改建绿化任务，建成睢阳公园、青年公园、包河游园、应天公园、民馨公园、南城湖公园、翠竹园、梅园和十块游园绿地精品升级改造。筹措资金3297万元，完成村（镇）绿化工程建设7．85万亩，建设“绿化生态示范村”1000个。

8．建成国家面粉及制品质量检验中心和食品药品检测中心。加强食品药品监管，全市城市、农村食品市场抽检合格率分别提高2个和3个百分点。

9．市10万吨污水处理厂力争年底建成并试运行。

10．商文化博物馆力争年底完成主体工程。

五、适应发展要求，推进服务型政府建设

新形势、新任务迫切要求政府全力加强自身建设，增强克难攻坚、应对挑战的行政能力，提高科学发展、改善民生的服务水平。

（一）坚持解放思想，增强服务意识。解放思想是克难致胜的法宝，越是在困难的时候越要解放思想，保持奋发有为的精神状态，探索科学有效方法，解决现实困难问题，努力闯出一片新天地。政府组成人员都要牢固树立为基层服务、为企业服务、为人民群众服务的思想，千方百计为企业排忧，满腔热情为群众解难，真心实意为基层办事。

（二）坚持科学决策，改进服务方式。一切从实际出发，按客观规律办事，涉及群众利益和公共政策的重大事项要广泛听取意见，做到问政于民、问需于民，尊重群众、顺应民意，努力避免决策失误，尽量做到少走弯路。强化责任意识，加快建立行政问责制，有责必问，有错必纠，切实维护人民群众的合法权益，规范约束行政行为，提高政府公信力。

（三）坚持依法行政，规范服务行为。全面贯彻落实《国务院关于加强市县政府依法行政的决定》，严格按照法定权限和程序行使权力，自觉接受人大的法律监督和政协的民主监督，认真办理人大代表建议和政协委员提案，加强与民主党派、工商联、无党派人士和各人民团体的联系，自觉接受公众监督和舆论监督。加强信息公开，推进电子政务，扩大政务公开，落实公民知情权、参与权、监督权。

（四）坚持求真务实，提高服务水平。坚持讲实话、办实事、求实效，恪尽职守，爱岗敬业，坚决反对官僚主义、形式主义，反对短期行为，反对虚报浮夸，努力创造经得起实践、人民和历史检验的工作实绩。发扬“严、细、深、实、快”的作风，精简文件、会议，改进文风、会风，集中精力多为群众解难题、办实事、谋福祉。

（五）坚持从严治政，强化服务保障。把反腐倡廉放在更加突出的位置，完善惩治和预防腐败体系，重点加强对建设工程招标投标、经营性土地使用权出让、房地产开发、政府投资项目管理等重点领域的监督。牢固树立过紧日子的思想，厉行节约，压缩开支，降低行政成本，确保今年会议、接待、出国、购车等公用经费零增长。加大专项治理力度，坚决查处、严肃处理“四乱”行为，为企业生产经营创造良好环境，切实纠正损害群众利益的不正之风。

各位代表！

困难激励斗志，耕耘收获果实，人民的信任和重托，是我们克难攻坚的强大动力。让我们高举中国特色社会主义理论伟大旗帜，在省委、省政府和市委的坚强领导下，坚持以科学发展观为指导，解放思想，抢抓机遇，同心同德，励精图治，奋力谱写商丘跨越发展的新篇章，以优异成绩向建国60周年献礼！
